# The BMI1 inhibitor PTC-209 is a potential compound to halt cellular growth in biliary tract cancer cells

**DOI:** 10.18632/oncotarget.6378

**Published:** 2015-11-24

**Authors:** Christian Mayr, Andrej Wagner, Magdalena Loeffelberger, Daniela Bruckner, Martin Jakab, Frieder Berr, Pietro Di Fazio, Matthias Ocker, Daniel Neureiter, Martin Pichler, Tobias Kiesslich

**Affiliations:** ^1^ Department of Internal Medicine I, Salzburger Landeskliniken – SALK, Paracelsus Medical University, Salzburg, Austria; ^2^ Laboratory for Tumor Biology and Experimental Therapies, Institute of Physiology and Pathophysiology, Paracelsus Medical University, Salzburg, Austria; ^3^ Research Program for Experimental Ophthalmology and Glaucoma Research, University Clinic of Ophthalmology and Optometry, Salzburger Landeskliniken – SALK, Paracelsus Medical University, Salzburg, Austria; ^4^ Laboratory of Functional and Molecular Membrane Physiology, Institute of Physiology and Pathophysiology, Paracelsus Medical University, Salzburg, Austria; ^5^ Department of Visceral, Thoracic and Vascular Surgery, Philipps-University Marburg, Marburg, Germany; ^6^ Institute for Surgical Research, Philipps-University Marburg, Marburg, Germany; ^7^ Present address: Experimental Medicine Oncology, Bayer Pharma AG, Berlin, Germany; ^8^ Present address: Department of Gastroenterology, Campus Benjamin Franklin, Charité University Medicine, Berlin, Germany; ^9^ Institute of Pathology, Salzburger Landeskliniken – SALK, Paracelsus Medical University, Salzburg, Austria; ^10^ Division of Oncology, Department of Internal Medicine, Medical University of Graz (MUG), Graz, Austria

**Keywords:** biliary tract cancer, PRC1, PTC-209, cell cycle arrest, BMI1

## Abstract

BMI1 is a core component of the polycomb repressive complex 1 (PRC1) and is up-regulated in biliary tract cancer (BTC), contributing to aggressive clinical features. In this study we investigated the cytotoxic effects of PTC-209, a recently developed inhibitor of BMI1, in BTC cells. PTC-209 reduced overall viability in BTC cell lines in a dose-dependent fashion (0.04 - 20 μM). Treatment with PTC-209 led to slightly enhanced caspase activity and stop of cell proliferation. Cell cycle analysis revealed that PTC-209 caused cell cycle arrest at the G1/S checkpoint. A comprehensive investigation of expression changes of cell cycle-related genes showed that PTC-209 caused significant down-regulation of cell cycle-promoting genes as well as of genes that contribute to DNA synthesis initiation and DNA repair, respectively. This was accompanied by significantly elevated mRNA levels of cell cycle inhibitors. In addition, PTC-209 reduced sphere formation and, in a cell line-dependent manner, aldehyde dehydrogease-1 positive cells. We conclude that PTC-209 might be a promising drug for future *in vitro* and *in vivo* studies in BTC.

## INTRODUCTION

The polycomb repressive complex 1 (PRC1) is a multi-subunit protein complex that represses gene transcription by mono-ubiquitylation of histone 2A at lysine 119 (H2AK119ub) [1]. Originally identified in *Drosophila melanogaster*, PRC1 is essential in diverse biological processes, including embryonic development, cell cycle progression, DNA repair, stem cells as well as apoptosis and senescence [1-3]. The core of the PRC1 complex is composed of a BMI, CBX, PHC, RING1 and RYBP subunit, of which RING1 exerts H2AK119ub activity. BMI is an essential co-factor for RING1 activity [4, 5]. Aberrant PRC1 activity contributes to cancer development in various types of tumors [6]. In particular, BMI1 is up-regulated in cancer and associated with general aggressiveness and cancer stem cells (CSC) [7-13].

Biliary tract cancer (BTC) is categorized in intrahepatic and extrahepatic cholangiocarcinoma (ICC, ECC), as well as gallbladder cancer (GBC) [14]. Although BTC is a rare malignancy in the US and Europe, mortality rates are very high due to late diagnosis and limited therapeutic options [15]. Recent studies suggest that overexpression of BMI1 drives tumorigenesis and progression of BTC (for a recent review see [5]). Two publications showed that BMI1 is up-regulated in BTC cell lines as well as in BTC patient samples [16, 17]. Additionally, it has been demonstrated that BMI1 is crucial for BTC cells to form colonies – an attribute associated with CSC [17]. In another study, immunostaining revealed that BMI1 was highly expressed in combined hepatocellular-cholangiocarcinoma and that BMI1 positivity correlated with enhanced expression of proliferation markers [18]. High expression levels of BMI1 were also seen in biliary intraepithelial lesions, precursors of CC, as well as in invasive CC. Interestingly, this observation was accompanied by declining levels of p16INK4A [19]. These data suggest that the PRC1 complex and especially BMI1 promote development and progression of BTC, making it an interesting therapeutic target.

Recently, Kreso et al. developed a small-molecule BMI1 inhibitor called PTC-209 and demonstrated its specific anti-tumor effects in colorectal cancer *in vitro* and *in vivo*. In particular, they showed that PTC-209 treatment reduces cancer cell growth and CSC properties [20]. Up to now, no data is available describing the effects of PTC-209 in BTC. Therefore, the current study investigated the effects of PTC-209 in BTC cells in order to evaluate PTC-209 as a potential drug for the treatment of BTC patients.

## RESULTS

### PRC core components are expressed in BTC cell lines

**Figure 1:**
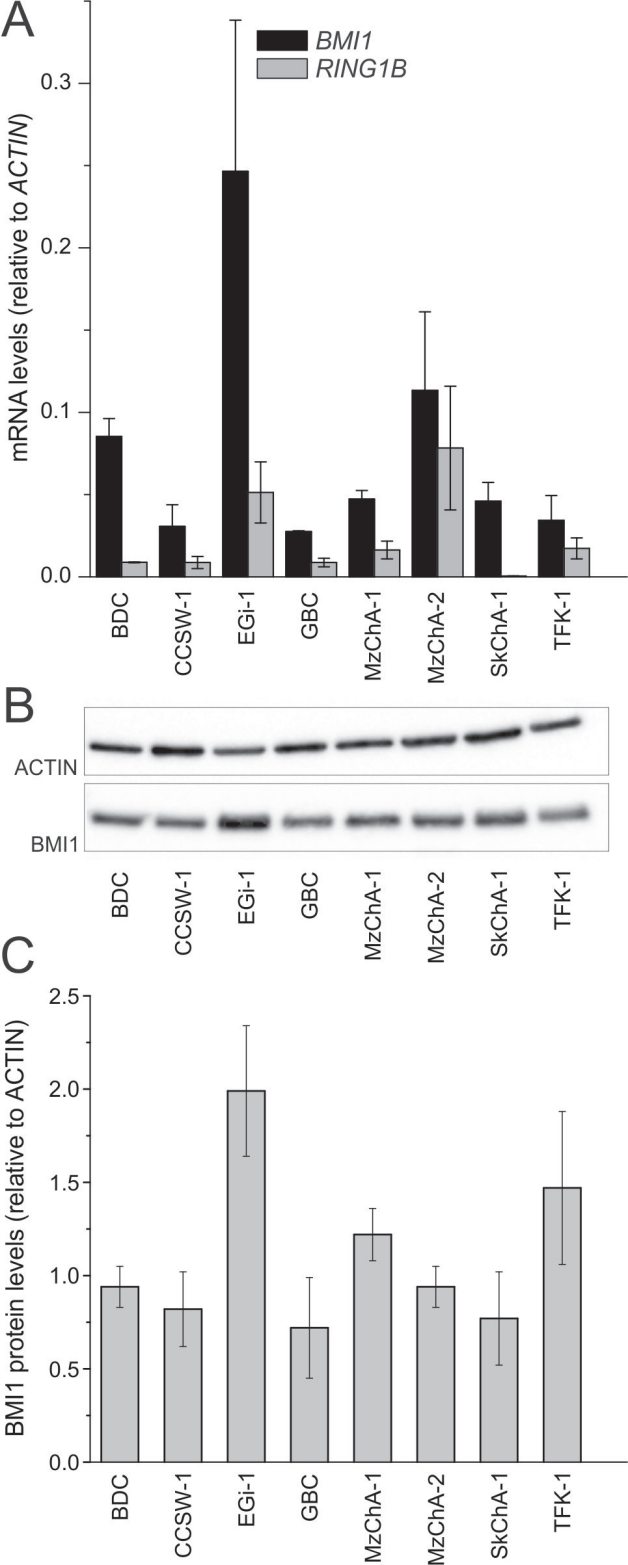
Expression of PRC1 components in BTC cell lines **A**. mRNA levels of PRC1 core components *BMI1* and *RING1B* in BTC cell lines (n = 3, n = 4 for EGi-1 and MzChA-2). **B**. Representative western blot image (cropped). **C**. Expression of BMI1 protein in BTC cell lines (n = ≥ 3). Abbreviations: BTC: biliary tract cancer; PRC1: polycomb repressive complex 1; BMI1: BMI1 polycomb ring finger oncogene; RING1B: ring finger protein 2.

The core components of the PRC1 complex *BMI1*/BMI1 and *RING1B* could be detected in all BTC cell lines at a various extent on mRNA level and/or protein level, respectively (Figure 1). Correlation analysis of mRNA and protein expression indicates a significant correlation (Pearson's correlation coefficient = 0.76, p=0.029) for these eight cell lines.

### PTC-209 inhibits proliferation of BTC cells

**Figure 2:**
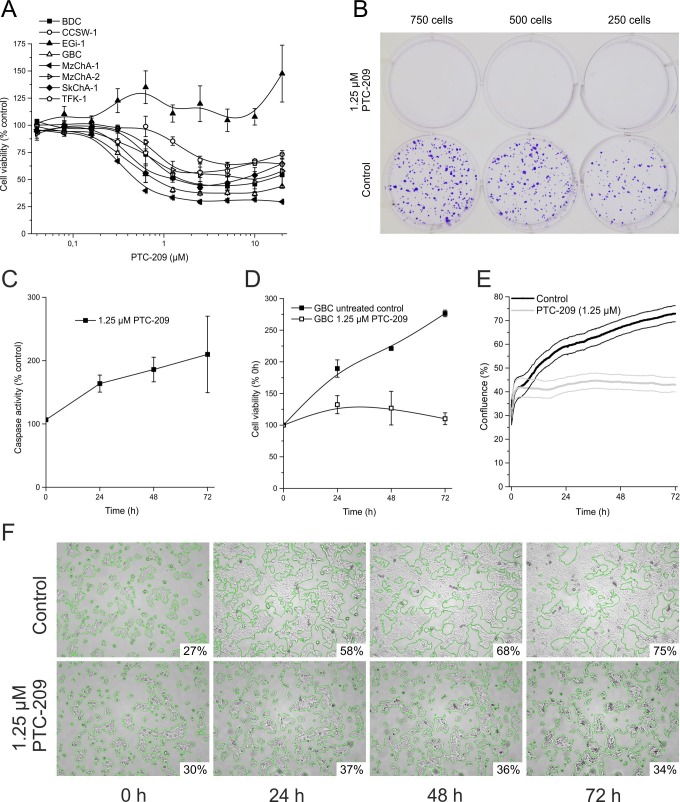
Cytotoxic effects of PTC-209 on BTC cells **A**. Dose-dependent effect of PTC-209 on cell viability of BTC cell lines after 72 h (n = 4, n = 6 for EGi-1). **B**. Clonogenic assay of GBC cells after 10 days (n = 4), image of representative assay. **C**. Time-dependent effect of 1.25 μM PTC-209 on caspase-3 and -7 activity in GBC cells (n = 4). **D**. Growth curve analysis of GBC cells treated with 1.25 μM PTC-209 for 72 h compared to untreated cells using standard cell viability assay (resazurin) (n = 4). **E**. Growth curve analysis of GBC cells treated with 1.25 μM PTC-209 for 72 h compared to untreated cells using the JuLi BR live cell movie analyzer (SEM error bars are indicated by encasing lines). Cell density of treated and untreated cells was measured in parallel every five minutes (865 images each) (n = 5). **F**. Representative images for GBC cells 0, 24, 48 and 72 h post PTC-209 treatment, respectively, generated by JuLi BR live cell movie analyzer. Green edges indicate the areas calculated for estimation of confluence (% of image section). See additional file 2 for a continuous video. Asterisks indicate significant (*, p<0.05) or highly significant (**, p<0.01) differences related to untreated control cells. Abbreviations: BTC: biliary tract cancer; h: hours.

The effect of PTC-209 on the overall cell viability of BTC cell lines after 72 h is shown in Figure 2A. PTC-209 significantly inhibited cell proliferation in a dose-dependent manner in seven of eight tested BTC cell lines (for significances and 10% or 50% inhibitory concentration (IC_10_, IC_50_) see additional file 1). There was no significant correlation between expression of *BMI1*/BMI1 and the effect of PTC-209 on the number of viable cells (IC_10_, p=0.8 and p=0.5 for mRNA and protein, respectively). GBC cells showed a significant response in all, but the two lowest PTC-209 concentrations and displayed low IC_10_ and IC_50_ values (additional file 1). Therefore, we used this cell line for all subsequent experiments.

To further analyze the effect of PTC-209 on proliferation and survival of BTC cells, clonogenic assays were performed, indicating that treatment of BTC cells with PTC-209 completely inhibited growth of clones (Figure 2B for GBC; see additional file 2 for other cell lines).

To evaluate whether these observations are caused by either growth arrest or a direct cytotoxic effect (e.g. apoptosis), we measured caspase activity in PTC-209-treated GBC cells. PTC-209 only slightly increased caspase activity (Figure 2C), indicating that the observed effects of PTC-209 on cell viability might be not primarily caused by induction of apoptosis, but rather by a stop of cellular growth.

To confirm this assumption, we analyzed cell growth using the resazurin viability assay and observed that 1.25 μM PTC-209 almost completely stopped cellular growth of GBC cells (Figure 2D). This observation was confirmed by an additional experiment in which we monitored the confluence as a surrogate marker of cellular growth in real-time by continuous live cell monitoring over 72 h. After calculation of the cell density for each picture, the cell growth curves (mean of five independent experiments) clearly show that PTC-209 impedes cellular growth (Figure 2E). Interestingly, this effect was already observable after 24 h of PTC-209 treatment as shown in Figure 2F (see additional file 3 for a representative video).

### PTC-209 causes cell cycle arrest

**Figure 3:**
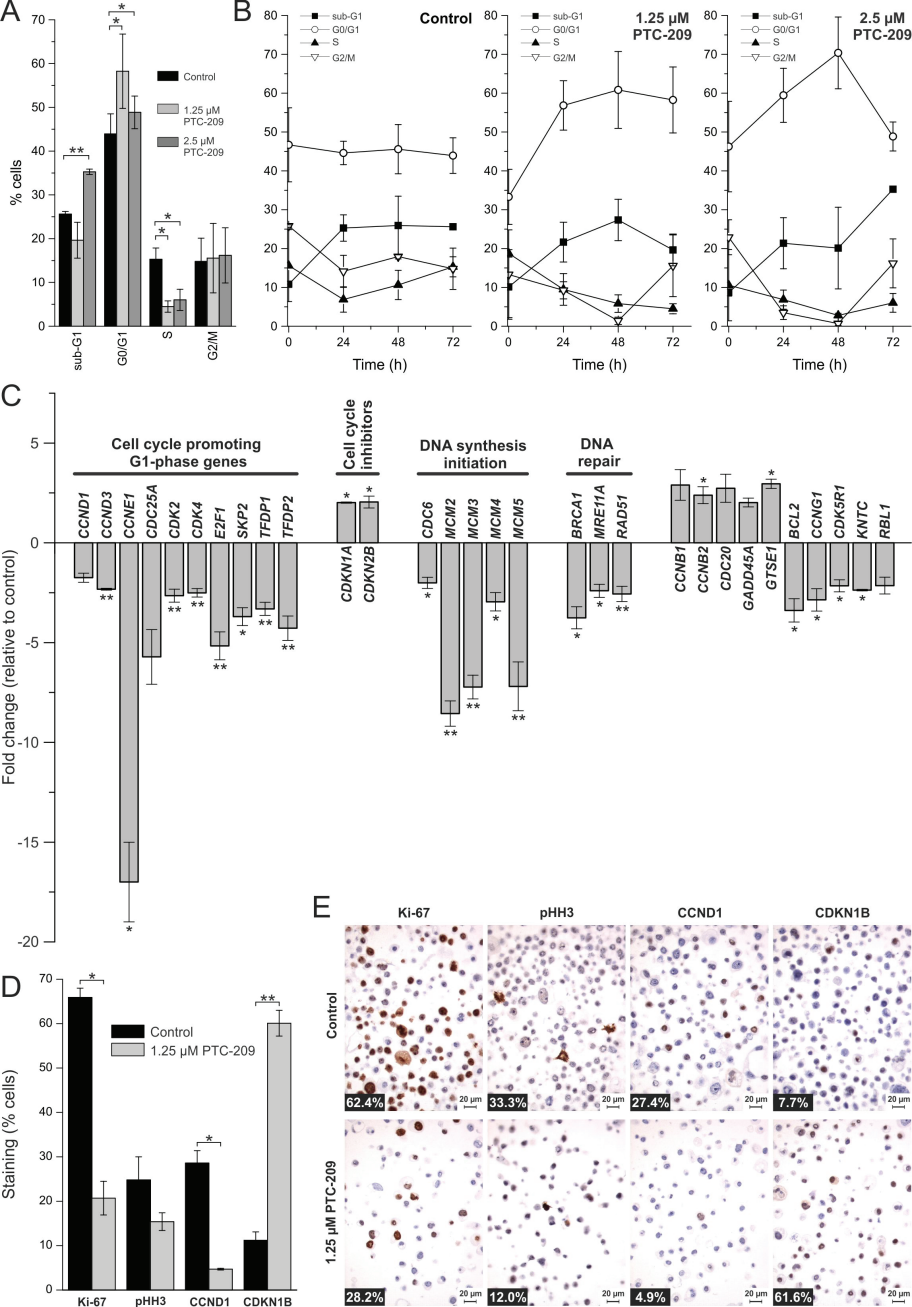
Effects of PTC-209 on the cell cycle **A**. Cell cycle distribution of PTC-209 treated GBC cells compared to untreated cells after 72 h (n = 4). **B**. Time-dependent effect of PTC-209 on the cell cycle distribution of GBC cells (n = 4). Sub-G1 represents cells with a DNA content less than 2N, G0/G1 cells with DNA content 2N, S cells with DNA content greater than 2N and G2/M cells with DNA content 4N. **C**. Changes in expression of cell cycle relevant genes after 24 h of 1.25 μM PTC-209 treatment (n = 4). Data were normalized to five housekeeping genes (*BETA-ACTIN*, *B2M*, *GAPDH*, *HPRT1* and *RPLP0*) according to the manufacturer's suggestion (Qiagen Data Analysis Center) and related to untreated controls. For full gene names and Refseq number see additional file 4. **D**. Quantitative analysis of immunostaining of GBC cells for Ki-67, pHH3, CCND1 and CDKN1B after 72 h (n = 3). **E**. Representative images of immunostaining of GBC cells. Numbers at bottom left corner indicate percentage of positive cells. Asterisks indicate significant (*, p<0.05) or highly significant (**, p<0.01) differences related to untreated control cells. Abbreviations: h: hours.

We next studied the effect of PTC-209 on the cell cycle in GBC cells. As shown in Figure 3A, 2.5 μM PTC-209 increased cells in sub-G1 (from 25% to 35%) after 72 h of incubation. Additionally, PTC-209 treatment caused a significant increase of cells in the G0/G1 phase, accompanied by a significant decrease of cells in the S phase after 72 h. The percentage of cells in the G2/M phase remained unchanged (Figure 3A). Time-resolved analysis of the effects of PTC-209 on the cell cycle (Figure 3B) indicates an increase of cells in the G0/G1 and sub-G1 phases and a decrease of cells in the S phase already 24 h after treatment.

To further investigate the effect of PTC-209 on the cell cycle, we performed a comprehensive qRT-PCR analysis covering changes in the mRNA levels of 84 cell cycle-related genes following 24 h of PTC-209 treatment. We considered genes with a fold change of greater than two as up- or down-regulated, respectively. Using this threshold, Figure 3C shows that PTC-209 treatment caused up-regulation of seven and a down-regulation of 24 genes (Figure 3C, for list and results of all 84 genes see additional file 4). Grouping of these genes shows that numerous cell cycle-promoting G1-phase genes were significantly down-regulated, including cyclins *CCND1*, *CCND3*, *CCNE1*, as well as *CDK-2/-4*, and *TFDP-1/-2*. These changes were accompanied by a significant up-regulation of two G1-phase inhibitors (*CDKN1A* and *CDKN2B*). Furthermore, we observed significant down-regulation of genes responsible for initiation of DNA synthesis (*CDC6*, *MCM2-5*). Additionally, PTC-209 reduced mRNA levels of DNA repair genes, such as *BRCA1*, *MRE11A*, and *RAD51*. In line with these observations, immunostaining revealed a reduction of cells positive for proliferation markers Ki-67, CCND1 and pHH3 after PTC-209 treatment (significant for Ki-67, CCND1; Figure 3D and 3E). Interestingly PTC-209 caused a significant increase of the cell cycle inhibitor CDKN1B (Figure 3D and 3E). This factor was also up-regulated on mRNA level by trend (fold change 1.7, see additional file 4) after PTC-209 treatment.

### PTC-209 reduces CSC subpopulations

**Figure 4:**
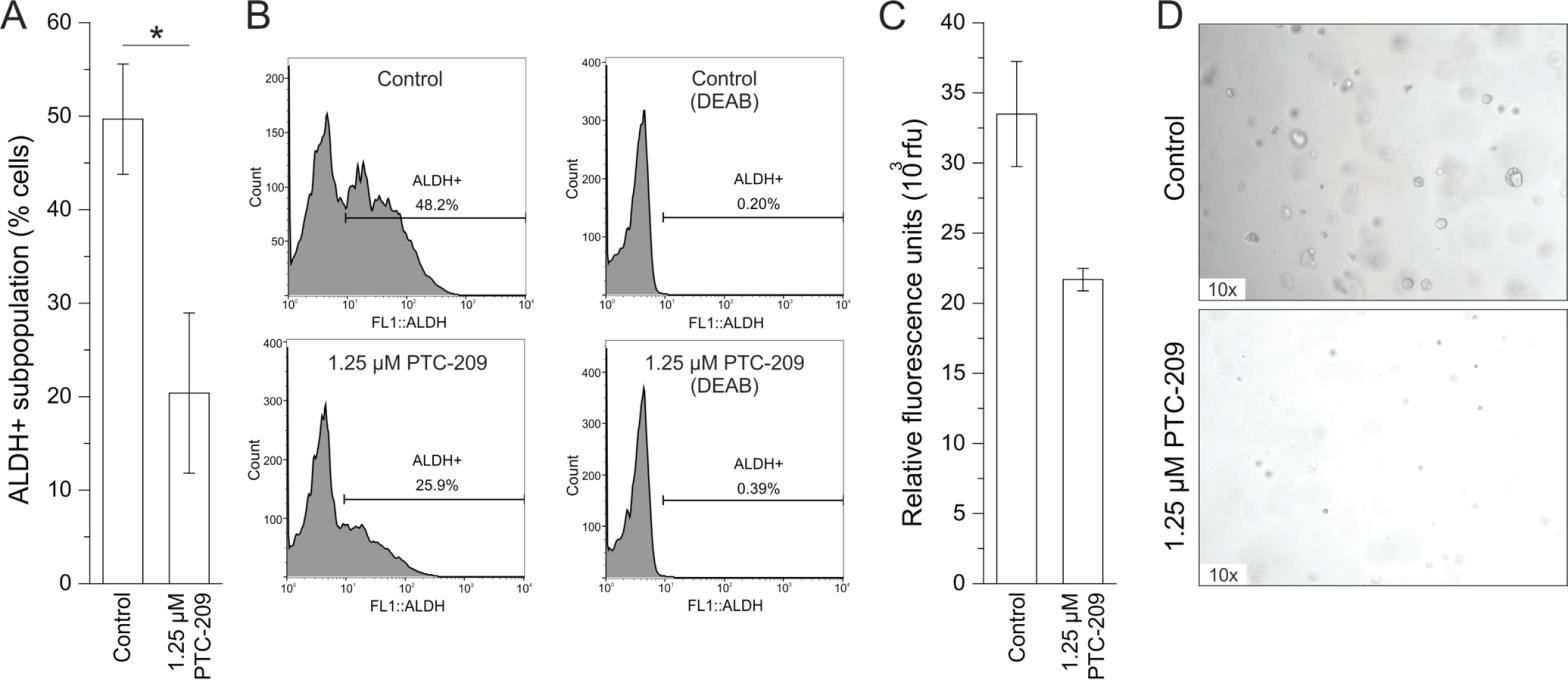
PTC-209 reduces functional stem cell characteristics **A**. Reduction of the ALDH+ subpopulation in GBC cells after 72 h of 1.25 μM PTC-209 treatment (n = 5). **B**. Example histograms for untreated and PTC-209 treated GBC cells (left) with corresponding negative DEAB controls (right). **C**. Reduction of sphere formation of GBC cells after 14 days of 1.25 μM PTC-209 treatment (n = 4). **D**. Representative images of sphere formation of GBC cells after 14 days. Asterisks indicate significant (*, p<0.05) differences related to untreated control cells. Abbreviations: ALDH+: aldehyde dehydrogenase-1 positive cells; DEAB: N,N-diethylaminobenzaldehyde; h: hours.

Treatment with PTC-209 caused a significant reduction of ALDH+ cells from 50% to 20% after 72 h in the GBC cell line (Figure 4A and 4B). It is worth mentioning that in two out of four experiments, PTC-209 completely abolished the ALDH+ subpopulation. However, this effect seems to be highly cell line-dependent, since PTC-209 treatment did not cause reduction of ALDH+ subpopulation in all eight BTC cell lines (see additional file 5). We additionally evaluated the effect of PTC-209 treatment on sphere formation of GBC cells as a second functional CSC marker. As shown in Figure 4C and 4D, PTC-209 reduced number and size of spheres *in vitro.*

### PTC-209 decreases protein levels of BMI1 and H2AK119ub

**Figure 5:**
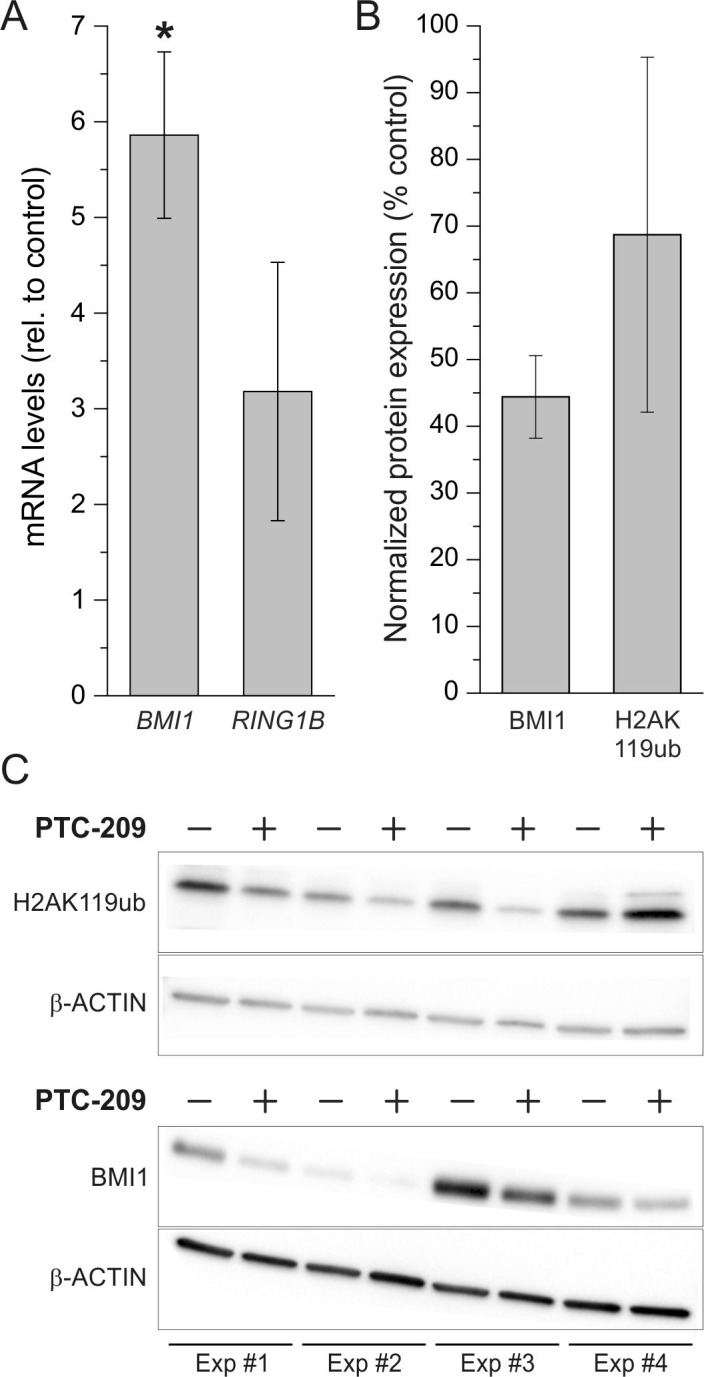
Effect of PTC-209 on mRNA expression of BMI1 and RING1B and on protein levels of BMI1 and H2AK119ub **A**. Changes of *BMI1* and *RING1B* mRNA levels after 72 h PTC-209 treatment (1.25 μM) in GBC cells. Data were normalized to *BETA-ACTIN* and related to untreated controls (n = 4 for *BMI1*, n = 3 for *RING1B*). **B**. Quantification of changes in protein levels of BMI1 and H2AK119ub in GBC cells after treatment with 1.25 μM PTC-209 for 72 h using the ImageJ software (n = 4). **C**. Western blot images of GBC cells treated with 1.25 μM PTC-209 for 72 h (n = 4, cropped). Abbreviations: BMI1: BMI1 polycomb ring finger oncogene; h: hours; H2AK119ub: mono-ubiquitylation of histone 2A at lysine 119; PRC1: polycomb repressive complex 1; RING1B: ring finger protein 2.

To evaluate whether the observed effects of PTC-209 in GBC cells are caused by an actual reduction of PRC1 activity we measured mRNA levels of *BMI1* and *RING1B* and protein levels of BMI1 and H2AK119ub, respectively, after treatment with PTC-209. Surprisingly, on mRNA level, treatment of GBC cells with PTC-209 caused an up-regulation *BMI1* and *RING1B* (Figure 5A). However, western blot analysis revealed a clear decline of BMI1 protein levels after PTC-209 treatment (Figure 5B and 5C). For H2AK119ub, PTC-209 treatment reduced protein levels in three out of four experiments (Figure 5B and 5C).

### PTC-209 shows potential synergy with cisplatin

**Figure 6:**
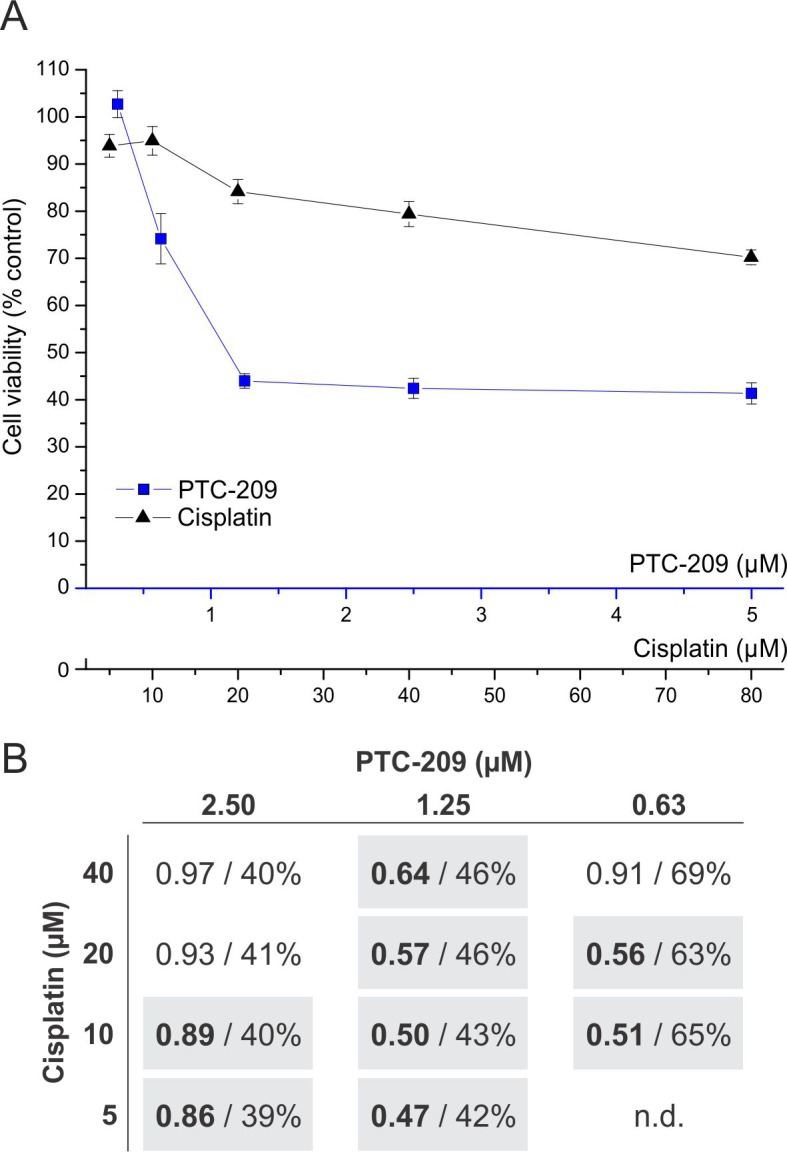
Synergistic cytotoxic effect of combined PTC-209 and cisplatin treatment **A**. Dose-dependent effect of single treatment with PTC-209 or cisplatin (n = 6). Blue line indicates viable cells after PTC-209 treatment, black line indicates viable cells after cisplatin treatment, respectively. **B**. Effect of combined PTC-209 and cisplatin treatment on GBC cells. Each cell represents a combination of specific concentrations of PTC-209 and cisplatin, respectively (n = 6). The first number (bold) indicates the CI and the second number shows the percentage of viable cells of the respective combination after 72 h compared to untreated cells. The calculation of the CI is based on the cell viability values of the single (A) and combined treatments. A CI smaller than 0.9 indicates a synergistic effect, whereas a CI between 0.9 and 1.1 represents an additive effect [22]. Synergistic combinations are highlighted with grey background. Abbreviations: CI: combination index; h: hours; n.d.: not determined.

Since treatment with PTC-209 reduced mRNA levels of genes involved in DNA repair (Figure 3C) we finally tested if a combination with the standard chemotherapeutic cisplatin [21] shows a synergistic effect – based on the mathematical model of Chou T.C. [22]. Although the absolute cytotoxicity values of the combined drugs are not considerably lower compared to PTC-209 alone, eight combinations of PTC-209 and cisplatin yielded a combination index (CI) below 0.9 indicating drug synergy (Figure 6).

## DISCUSSION

In this study we provide first data on the anti-cancer effects of PTC-209 in BTC cell lines: i) treatment of eight different BTC cell lines with various concentrations of PTC-209 significantly inhibited number of viable cells in seven cell lines, ii) this effect is mainly mediated by a stop of cell growth, iii) cell cycle and gene expression analysis of cell cycle-related genes confirm a cell cycle arrest at G1/S, iv) PTC-209 may inhibit putative CSC as it reduces sphere formation and the amount ALDH+ BTC cells (cell line-dependent), and, v) combined treatment with cisplatin shows a synergistic effect for several combinations.

For one cell line (EGi-1), the number of viable cells was not reduced compared to untreated controls. This is surprising, since EGi-1 showed the highest expression of *BMI1* on mRNA level and also high expression of BMI1 protein. The reasons remain speculative, but genetic alterations of the BMI1 gene or downstream genes might explain the non-responsiveness of this cell line. Since all other seven BTC cell lines used in this study showed significant responsiveness for PTC-209, future projects need to investigate the underlying mechanisms of resistance to identify potential biomarkers for PTC-209 sensitive tumors.

While the anti-cancer effects of PTC-209 were mediated by cell cycle exit and apoptosis induction in colorectal tumor-initiating cells [20], the cytotoxic effects of PTC-209 in the investigated BTC cells were rather caused by an inhibition of cell growth than apoptosis. Following PTC-209 treatment, we saw an accumulation of cells in the G0/G1 phase of the cell cycle, accompanied by a significant reduction of cells in the S-phase, indicating a cell cycle stop at the G1/S checkpoint. Interestingly, this effect was already observable after 24 h of PTC-209 treatment. This observation goes in line with findings by Ismail et al., which describe that PRC1 inhibition led to reduction of ubiquitylated H2A as early as one hour after treatment [23]. Additionally, immunostaining revealed a decline of cells positively stained for proliferation markers Ki-67, pHH3 and CCND1 (significant for Ki-67 and CCND1), accompanied by a significant increase of the cell cycle inhibitor CDKN1B.

**Figure 7:**
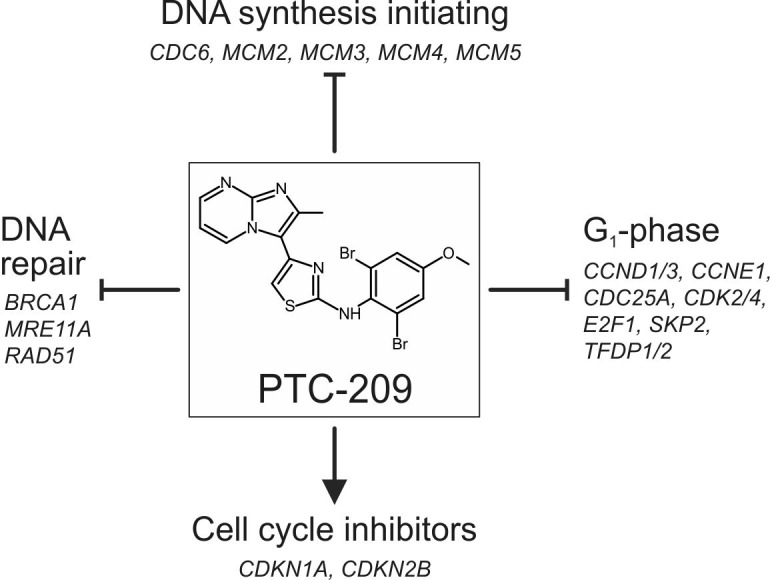
Summarized effects of PTC-209 on the cell cycle in biliary tract cancer cells PTC-209 may inhibit cell cycle progression by down-regulation of genes involved in DNA synthesis initiation, DNA repair and cell cycle promotion at G_1_ accompanied by up-regulation of cell cycle inhibitors – for details see Figure 3C. For full gene names and Refseq numbers see additional file 4.

To provide first information on the mechanism of action of PTC-209 causing cell cycle stop in BTC cells, we comprehensively analyzed changes in expression of cell cycle-related genes after PTC-209 treatment (see Figure 7 for summary). PTC-209 significantly reduced the expression of numerous genes that promote cell cycle in the G1-phase. To our current understanding, the CCND/CDK4 complex activates E2F-1, which in turn leads to the transcription of its target genes, including itself, CCNE and CDC25a. CCNE then associates with CDK2 to control G1 progression [24]. PTC-209 caused a significant mRNA up-regulation of the two cell cycle inhibitors *CDKN1A* (inhibits CCND/CDK4) and *CDKN2B* (inhibits CCNE/CDK2). Additionally, PTC-209 diminished mRNA levels of *TFDP1* and *TFDP2*, which are known to form complexes with E2F-1 to enhance its transcriptional activity [25].

SKP2 has been shown to be important for progression of the cell cycle in the G1-phase [26, 27] and PTC-209 treatment caused a significant down-regulation of *SKP2* in our study. Interestingly, a recent publication has correlated SKP2 expression in extrahepatic CC with enhanced proliferation and poorer survival and identified SKP2 as an independent prognostic marker [28]. The same study also suggests SKP2 as a potential target of E2F-1 – a gene that was significantly down-regulated after PTC-209 treatment in our study.

An important step for cell cycle progression is the initiation of DNA synthesis. During the G1-phase of the cell cycle, the assembly of the pre-replication complex starts by recruitment of CDC6 to the origin of replication [29]. This allows binding of MCM2-7, a complex of six highly conserved proteins that ensures proper DNA replication [29]. When treated with PTC-209, *CDC6* as well as four *MCM* genes (2-5) were significantly down-regulated, suggesting that PTC-209 may inhibit initiation of DNA replication. Interestingly, a study by Bracken and coworkers showed that siRNA-mediated knockdown of *BMI1* resulted in diminished expression levels of *CDC6* [30], making *CDC6* a potential BMI1 target. Increased expression of *MCM* genes has been found in various types of cancer and has been associated with short survival rates [31-33]. In gallbladder cancer, MCM2 has been shown to serve as an independent predictor of poor outcome, since expression of MCM2 was correlated with larger tumor mass, poor differentiation, lymph node metastasis and shorter survival [34].

While insufficient DNA damage repair can lead to the development of cancer in the first place, DNA damage repair is one mechanism of cancer cells to develop therapeutic resistance [21, 35, 36]. Therefore, inhibition of the DNA repair machinery in cancer cells is a desired effect, especially when combined with DNA damaging therapies. Following PTC-209 treatment, we observed decreased mRNA levels of *BRCA1*, *MRE11A* and *RAD51*, genes that are involved in homology-directed DNA repair [36]. One potential underlying mechanism by which PTC-209 may inhibit DNA damage repair includes the RAD51 interacting protein RAD51AP [37], which is also known to be an E2F-1 target [38]. By down-regulation of *E2F-1*, PTC-209 may indirectly also diminish transcription levels of *RAD51AP*, which in turn (and combined with the reduced *RAD51* mRNA levels caused by PTC-209 treatment) might impair RAD51 activity. Interestingly, *RAD51AP* was related to therapeutic resistance in colon cancer cells, underlining its importance as a potential therapeutic target [39]. Concerning BTC, Obama et al. demonstrated, that RAD51AP was up-regulated in intrahepatic CC and associated with proliferation, making it also a potential candidate for BTC treatment [40]. In this regard we asked the question if treatment with PTC-209 can potentially augment chemotherapy in BTC. Based on the calculation of the combination index (CI) [22], several combinations of PTC-209 with the standard chemotherapeutic cisplatin showed a synergistic cytotoxic effect, making PTC-209 an interesting adjuvant drug for future more detailed studies using chemoresistant BTC cells.

In the initial study by Kreso et al., PTC-209 treatment caused apoptosis and cell cycle exit in colorectal cancer-initiating cells, i.e. CSC [20]. There is strong evidence that CSC drive tumorigenesis in BTC [41-45]. In the current study we investigated the effect of PTC-209 on two functional stem cell characteristics, ALDH positivity and sphere formation [46-50]: PTC-209 reduced the ALDH+ subpopulation in four cell lines (GBC, CCSW, MzChA-2 and TFK-1), whereas in the remaining four cell lines the ALDH+ subpopulation was not affected or slightly increased (BDC, EGi-1, MzChA-1 and SkChA-1), suggesting a strong cell line-dependence. Interestingly, Shuang et al. demonstrated that the ALDH+ subpopulation in the BTC cell line TFK-1 had reduced sensitivity to 5-fluorouracil and showed higher proliferation potential. In patients, expression of ALDH1 was a predictor for poor prognosis [45]. Additionally, we recently demonstrated that the ALDH+ subpopulation in BTC cells highly expresses known stem cell markers compared to ALDH negative cells [51]. Furthermore, in the present study we observed reduced sphere formation of BTC cells *in vitro* after PTC-209 treatment, underlining the potential effect of PTC-209 on CSC characteristics. This goes in line with Kreso et al., who showed that PTC-209 significantly diminished sphere-initiating cell frequency in colorectal cancer samples [20].

While PTC-209 caused an up-regulation of *BMI1* on mRNA level, BMI1 protein levels were clearly reduced after PTC-209 treatment. This phenomenon may be explainable by an overcompensation reaction of the cells on the mRNA level. As treatment with PTC-209 resulted in reduced ubiquitylation of H2AK119 as the effective histone modification in three out of four experiments, we conclude that the mechanism of PTC-209 involves BMI1 as a direct target – in agreement with the initial study by Kreso et al. [20].

In summary, the actual study provides for the first time evidence for a significant anti-cancer activity of PTC-209 in BTC cells. *In vitro,* PTC-209 treatment causes cell cycle arrest in BTC cells and cell line-dependent reduction of functional CSC characteristics. The present data suggests that PTC-209 is a potential anti-cancer agent for BTC – provided that subsequent future studies yield conclusive data on its anti-cancer efficiency and possible side effects in an *in vivo* BTC model, its bioavailability in humans and possible mechanisms responsible for drug sensitivity.

## MATERIALS AND METHODS

### Substances and cell culture

PTC-209 was purchased from Selleckchem (Houston, Texas, USA), dissolved in dimethyl sulfoxide (Sigma Aldrich, Vienna, Austria) at a stock concentration of 10 mM and stored in aliquots at −20°C. Resazurin was obtained from Sigma Aldrich and dissolved in Dulbecco's Phosphate Buffered Saline (DPBS, Sigma Aldrich). For our experiments, five bile duct carcinoma cell lines BDC (G4, [52]), CCSW-1 (G2, [53]), EGi-1 (G3, [54]), SkChA-1 (G3, [55]), TFK-1 (G2, [56]) and three gallbladder cancer cell lines GBC (G1, [57]), MzChA-1 (G1, [55]) and MzChA-2 (G2, [55]) were used and termed together as BTC cell lines [58]. The cells were cultured in high glucose Dulbecco's modified Eagle's medium (DMEM; Gibco, Life Technologies, Vienna, Austria) supplemented with 10% (v/v) fetal bovine serum (FBS; Gibco, Life Technologies) as described before [59, 60]. Cells were seeded using the following concentrations per cm^2^ of the cell culture receptacle in 10% FBS DMEM: 3.95*10^4^ (BDC, MzChA-2), 4.74*10^4^ (CCSW-1, GBC), 5.53*10^4^ (SkChA-1), 6.32*10^4^ (EGi-1, TFK-1), and 7.11*10^4^ (MzChA-1). For PTC-209 drug treatment, we used serum-free DMEM (sfDMEM) to avoid possible interactions of the drugs with components of the serum.

### Drug cytotoxicity

Dose-dependent cytotoxicity of PTC-209 for all eight BTC cell lines was investigated on cells grown in 96-well microplates. A dilution series of PTC-209 (0.04 - 20 μM) was applied for 72 h in sfDMEM. Quantification of cell viability was done using the resazurin assay and an Infinite M200 microplate reader (Tecan, Groedig, Austria) as described previously [59, 61]. Viability was related to untreated control (sfDMEM-only) samples. For time course experiments, GBC cells were seeded in 96-well microplates and 1.25 μM PTC-209 in sfDMEM were added. Time points were taken at 0 h, 24 h, 48 h and 72 h, respectively, and viability was measured using the resazurin assay and an Infinite M200 microplate reader. Viability was related to the initial 0 h time point. Additional time course experiments were carried out on the JuLi BR live cell movie analyzer (NanoEnTek, Seoul, Korea). Here, GBC cells were seeded in 30 mm cell culture dishes and either treated with 1.25 μM PTC in sfDMEM or left untreated (sfDMEM only). Cell growth was monitored in parallel on two microscopy units in a humidified cell culture incubator (37°C, 5% CO_2_) by taking brigthfield microscopy images every five minutes for 72 h (865 images per sample). The built-in software calculated the confluence (% image area) for each image allowing for continuous monitoring of the cell growth. For apoptosis analysis, the Caspase-Glo® 3/7 Assay (Promega, Mannheim, Germany) was used, according to manufacturer's protocol in 96-well microplates. Measurement of caspase-3 and -7 activity was performed on an Infinite M200 microplate reader. Caspase activity was related to corresponding control (sfDMEM only) samples for each time point.

### Drug synergy

For combined PTC-209 and cisplatin treatment, GBC cells were seeded in 96-well microplates and treated with different concentrations of each drug alone as well as different combinations of PTC-209 and cisplatin (single treatments for PTC-209: 5 μM, 2.5 μM, 1.25 μM, 0.63 μM and 0.31 μM; single treatments for cisplatin: 80 μM, 40 μM, 20 μM, 10 μM and 5 μM – see Figure 6A; combinations: 40 μM cisplatin with 2.5, 1.25 or 0.63 μM PTC-209 respectively; 20 μM cisplatin with 2.5, 1.25 or 0.63 μM PTC-209 respectively; 10 μM cisplatin with 2.5, 1.25 or 0.63 μM PTC-209 respectively and 5 μM cisplatin with 2.5 or 1.25 μM respectively – see Figure 6B). To ensure validity of the experiments, each individual experiment (containing all single and combination treatments) was performed on one 96-well microplate. To evaluate a possible synergistic cytotoxic effect, the combination index (CI) was calculated using the CompuSyn software (www.combosyn.com) as described before [22, 62]. Concentrations that resulted in CI values less than 0.9 were considered as synergistic, whereas CI values between 0.9 and 1.1 were considered as additive [22].

### Clonogenic assay

Cells were seeded in 6-well plates using 2000, 1500 and 1000 cells per well for CCSW-1 and TFK-1 and 750, 500 and 250 cells per well for BDC, EGi-1, GBC, MzChA-1, MzChA-2 and SkChA-1. Cells were left untreated (sfDMEM only) or treated with 1.25 μM PTC-209 for 7 to 14 days, dependent on the cell line. Clones were fixed and stained with staining solution (80% PBS, 20% methanol (Sigma Aldrich), 0.1% crystal violet (Sigma Aldrich)) for 20 minutes at room temperature, air-dried overnight and photographed using a Panasonic DMC-FZ28 camera.

### Immunostaining

GBC cells were seeded in 100 mm cell culture dishes and were either left untreated (sfDMEM only) or treated with 1.25 μM PTC-209 for 72 h. After harvesting and resuspension with 200 μl citrate plasma (supplied by the Institute of Transfusion Medicine, Paracelsus Medical University, Salzburg, Austria) and coagulation with 200 μl Thromborel® S (Siemens Healthcare Diagnostics, Deerfield, Illinois, USA) the clumped cells were fixed for 1 h in neutral-buffered saline containing 7% formalin and then paraffin-embedded as described before [60].

Cell blocks were cut into 5 μm sections and deparaffinized using graded alcohols. Antigen retrieval was performed by heat-induced epitope retrieval in pH 9 antigen retrieval buffer (Dako, Glostrup, Denmark) at 95°C for 1 h. Endogenous peroxidase blocking was carried out for 10 minutes with peroxidase blocking reagent (Dako). Subsequently, the primary antibodies against Ki-67 (mouse monoclonal, Clone MIB-1, dilution 1:500, Dako), pHH3 (rabbit polyclonal, dilution 1:200, Cell Marque, Rocklin, California, USA), CCND1 (rabbit monoclonal, Clone SP4, dilution 1:50, Neomarkers/Thermo Fisher Scientific, Waltham, Massachusetts, USA) and CDKN1B (mouse monoclonal, Clone SX53G8, dilution 1:100, Dako) were applied for 30 minutes at room temperature. The mouse or rabbit linker (Dako) was used for signal enhancement. Detection and visualization was performed using the Envision™ Flex Kit (Dako) based on the chromogen substrate diaminobenzidine (Roche Molecular Biochemicals, Mannheim, Germany) according to the manufacturer's instructions. Slides were counterstained with hematoxylin. The immunhistochemical stainings were done on the Autostainer Plus (Dako) platform. The stained slides were digitalized using the IMS (version v14q3, Imagic Bildverarbeitung, Glattbrugg, Switzerland) and analyzed using Image J (NIH, USA).

### Gene expression analysis

Total RNA was isolated with TRIzol Reagent (Ambion / Life Technologies, Vienna, Austria) and the Direct-zol™ RNA MiniPrep Kit (Zymo Research, Irvine, California, USA) followed by cDNA synthesis using the ImProm-II™ Reverse Transcription System (Promega). Gene expression was quantified by quantitative real-time reverse transcription PCR (qRT-PCR) using GoTaq qPCR Master Mix (Promega) on a ViiA7 real-time PCR system (Applied Biosystems, Life Technologies). All samples were measured at least in biological triplicates and checked for specificity of primer pairs by melting curve analysis. For calculation, samples were normalized to *BETA-ACTIN* as the housekeeping gene. PTC-209-treated samples were related to untreated controls according to the ΔΔCt method [63]. Primer sequences for qRT-PCR are as follows: *BMI1*-fwd: ATCCTTCTGCTGATGCTGCCAA, *BMI1*-rev: CACCTCCTCCTTAGATTTCTCTTTGTCTT, *RING1B*-fwd: CTCAGGAGGCAATAACAGATGGCTT, *RING1B*-rev: GACATTCTTTGTTGCCACTTCTAAGGG, *BETA-ACTIN*-fwd: GCACTCTTCCAGCCTTCCTTCC, and *BETA-ACTIN*-rev: TCTTTGCGGATGTCCACGTCAC. Expression changes of cell cycle relevant genes after 24 h of PTC-209 treatment of GBC cells were investigated with the RT^2^ Profiler PCR Array (Qiagen, Hilden, Germany) according to manufacturer's protocol and a ViiA7 real-time PCR system. For data analysis and sample normalization, Qiagen's Data Analysis Center was used. All samples were measured in biological triplicates and checked for specificity of primer pairs by melting curve analysis. Samples were normalized to commonly used housekeeping genes (*BETA-ACTIN*, *B2M*, *GAPDH*, *HPRT1* and *RPLP0*). Up-regulated genes (fold change greater than two) are represented as ΔΔCt values related to untreated controls, down-regulated genes (fold change less than two) are represented as –(1/fold change) related to untreated controls according to the Qiagen Data Analysis Center.

### Protein analysis

Cells without treatment or after incubation with 1.25 μM PTC-209 (72 h) were harvested by trypsin-EDTA treatment and counted. After centrifugation at 400 x g (3 minutes), the cell pellet was resupended in DPBS. Then cells were sonified (7-10 pulses, Sonopuls HD70, Bandelin, Berlin, Germany) and incubated for 5 minutes at 95°C in 2x SDS sample buffer. After that, 10^5^ cells per sample were loaded on SDS gels (4-20% Mini-PROTEAN TGX, BioRad, Vienna, Austria). Western blot was performed with the Trans-Blot Turbo Mini Nitrocellulose Transfer Packs System (BioRad). Antibodies were used with the following concentrations: BMI1 (1:1000, Cell Signaling Technology, Danvers, Massachusetts, USA), H2AK119ub (1:300, Diagenode, Seraing, Belgium), BETA-ACTIN (1:1000, Cell Signaling Technology) and anti-rabbit IgG, HRP-linked (1:1000, Cell Signalling Technology). Membranes were developed with Signal Fire ECL Reagent (Cell Signaling Technology) and protein bands were detected and quantified using a ChemiDoc MP System (BioRad) and Image J, respectively.

### Aldehyde dehydrogenase-1 assay

The effect of PTC-209 on the ALDH+ CSC subpopulation was examined using the ALDEFLUOR™ Kit (Stemcell Technologies, Grenoble, France) according to the manufacturer's protocol. Cells were either treated with 1.25 μM PTC-209 in sfDMEM or left untreated in sfDMEM for 72 h. Quantification of the ALDH+ subpopulation was done on a CellLab Quanta SC flow cytometer (Beckman Coulter, Krefeld, Germany). For calculation, the FlowJo software (Ashland, Oregon, USA) was used [64].

### Sphere formation assay

Anchorage-independent growth of GBC cells was examined using the CytoSelect 96-well Cell Transformation Assay (Cell Biolabs, San Diego, California, USA) and an Infinite M200 microplate reader. GBC cells were seeded at a concentration of 5*10^5^ cells per ml and incubated with 1.25 μM PTC-209 or left untreated for 10-12 days. Sphere formation was quantified with the CyQuant GR Dye (Cell Biolabs) according to the manufacturer's protocol. Visual analysis of anchorage-independent growth was done using an inverse microscope Axio ObserverZ1 (Zeiss, Göttingen, Germany).

### Cell cycle analysis

The distribution within the phases of the cell cycle was investigated by flow cytometry with ethanol-fixed, propidium iodide stained cells (staining solution: 0.04 mg/ml propidium iodide (Sigma Aldrich) and 0.1 mg/ml RNase (Ribonuclease A from bovine pancreas, Sigma Aldrich)) in DPBS after treatment with 1.25 μM or 2.5 μM PTC-209 for 0, 24, 48 and 72 h, respectively on a Quanta SC flow cytometer. For data analysis, FlowJo software was used.

### Statistics

All data points represent mean values of at least three biological replicates (n ≥ 3) ± SEM. Paired student's t-test was used for calculation of significance between groups. All calculations were performed using OriginPro 9.1 (OriginLab, Northampton, Massachusetts, USA). Statistical results were considered significant (*) or highly significant (**) at p<0.05 and p<0.01, respectively.

## SUPPLEMENTARY MATERIAL





## References

[1] Sauvageau M, Sauvageau G (2010). Polycomb group proteins: multi-faceted regulators of somatic stem cells and cancer. Cell stem cell.

[2] Luis NM, Morey L, Di Croce L, Benitah SA (2012). Polycomb in stem cells: PRC1 branches out. Cell stem cell.

[3] Park IK, Morrison SJ, Clarke MF (2004). Bmi1, stem cells, and senescence regulation. The Journal of clinical investigation.

[4] Cao R, Tsukada Y, Zhang Y (2005). Role of Bmi-1 and Ring1A in H2A ubiquitylation and Hox gene silencing. Molecular cell.

[5] Mayr C, Neureiter D, Wagner A, Pichler M, Kiesslich T (2014). The role of polycomb repressive complexes in biliary tract cancer. Expert opinion on therapeutic targets.

[6] Siddique HR, Saleem M (2012). Role of BMI1, a stem cell factor, in cancer recurrence and chemoresistance: preclinical and clinical evidences. Stem Cells.

[7] van Leenders GJ, Dukers D, Hessels D, van den Kieboom SW, Hulsbergen CA, Witjes JA, Otte AP, Meijer CJ, Raaphorst FM (2007). Polycomb-group oncogenes EZH2, BMI1, and RING1 are overexpressed in prostate cancer with adverse pathologic and clinical features. European urology.

[8] Hayry V, Tynninen O, Haapasalo HK, Wolfer J, Paulus W, Hasselblatt M, Sariola H, Paetau A, Sarna S, Niemela M, Wartiovaara K, Nupponen NN (2008). Stem cell protein BMI-1 is an independent marker for poor prognosis in oligodendroglial tumours. Neuropathology and applied neurobiology.

[9] Wang H, Pan K, Zhang HK, Weng DS, Zhou J, Li JJ, Huang W, Song HF, Chen MS, Xia JC (2008). Increased polycomb-group oncogene Bmi-1 expression correlates with poor prognosis in hepatocellular carcinoma. Journal of cancer research and clinical oncology.

[10] Liu JH, Song LB, Zhang X, Guo BH, Feng Y, Li XX, Liao WT, Zeng MS, Huang KH (2008). Bmi-1 expression predicts prognosis for patients with gastric carcinoma. Journal of surgical oncology.

[11] Vrzalikova K, Skarda J, Ehrmann J, Murray PG, Fridman E, Kopolovic J, Knizetova P, Hajduch M, Klein J, Kolek V, Radova L, Kolar Z (2008). Prognostic value of Bmi-1 oncoprotein expression in NSCLC patients: a tissue microarray study. Journal of cancer research and clinical oncology.

[12] Proctor E, Waghray M, Lee CJ, Heidt DG, Yalamanchili M, Li C, Bednar F, Simeone DM (2013). Bmi1 enhances tumorigenicity and cancer stem cell function in pancreatic adenocarcinoma. PloS one.

[13] Abdouh M, Facchino S, Chatoo W, Balasingam V, Ferreira J, Bernier G (2009). BMI1 sustains human glioblastoma multiforme stem cell renewal. The Journal of neuroscience : the official journal of the Society for Neuroscience.

[14] Razumilava N, Gores GJ (2014). Cholangiocarcinoma. Lancet.

[15] Patel T (2011). Cholangiocarcinoma--controversies and challenges. Nature reviews Gastroenterology & hepatology.

[16] Kemmerling R, Alinger B, Dietze O, Bosmuller HC, Ocker M, Wolkersdorfer GW, Berr F, Neureiter D, Kiesslich T (2012). Association of stem cell marker expression pattern and survival in human biliary tract cancer. International journal of oncology.

[17] Sasaki M, Yamaguchi J, Ikeda H, Itatsu K, Nakanuma Y (2009). Polycomb group protein Bmi1 is overexpressed and essential in anchorage-independent colony formation, cell proliferation and repression of cellular senescence in cholangiocarcinoma: tissue and culture studies. Human pathology.

[18] Sasaki M, Ikeda H, Itatsu K, Yamaguchi J, Sawada S, Minato H, Ohta T, Nakanuma Y (2008). The overexpression of polycomb group proteins Bmi1 and EZH2 is associated with the progression and aggressive biological behavior of hepatocellular carcinoma. Laboratory investigation; a journal of technical methods and pathology.

[19] Sasaki M, Yamaguchi J, Itatsu K, Ikeda H, Nakanuma Y (2008). Over-expression of polycomb group protein EZH2 relates to decreased expression of p16 INK4a in cholangiocarcinogenesis in hepatolithiasis. The Journal of pathology.

[20] Kreso A, van Galen P, Pedley NM, Lima-Fernandes E, Frelin C, Davis T, Cao L, Baiazitov R, Du W, Sydorenko N, Moon YC, Gibson L, Wang Y, Leung C, Iscove NN, Arrowsmith CH (2014). Self-renewal as a therapeutic target in human colorectal cancer. Nature medicine.

[21] Valle J, Wasan H, Palmer DH, Cunningham D, Anthoney A, Maraveyas A, Madhusudan S, Iveson T, Hughes S, Pereira SP, Roughton M, Bridgewater J (2010). Cisplatin plus gemcitabine versus gemcitabine for biliary tract cancer. N Engl J Med.

[22] Chou TC (2006). Theoretical basis, experimental design, and computerized simulation of synergism and antagonism in drug combination studies. Pharmacol Rev.

[23] Ismail IH, McDonald D, Strickfaden H, Xu Z, Hendzel MJ (2013). A small molecule inhibitor of polycomb repressive complex 1 inhibits ubiquitin signaling at DNA double-strand breaks. The Journal of biological chemistry.

[24] Berridge MJ (2012). Cell Cycle and Proliferation. Cell Signalling Biology.

[25] Di Fiore R, D'Anneo A, Tesoriere G, Vento R (2013). RB1 in cancer: different mechanisms of RB1 inactivation and alterations of pRb pathway in tumorigenesis. Journal of cellular physiology.

[26] Carrano AC, Eytan E, Hershko A, Pagano M (1999). SKP2 is required for ubiquitin-mediated degradation of the CDK inhibitor p27. Nature cell biology.

[27] Nakayama K, Nagahama H, Minamishima YA, Miyake S, Ishida N, Hatakeyama S, Kitagawa M, Iemura S, Natsume T, Nakayama KI (2004). Skp2-mediated degradation of p27 regulates progression into mitosis. Developmental cell.

[28] Kim JY, Kim HJ, Park JH, Park DI, Cho YK, Sohn CI, Jeon WK, Kim BI, Kim DH, Chae SW, Sohn JH (2014). Epidermal growth factor upregulates Skp2/Cks1 and p27(kip1) in human extrahepatic cholangiocarcinoma cells. World journal of gastroenterology : WJG.

[29] Lei M, Tye BK (2001). Initiating DNA synthesis: from recruiting to activating the MCM complex. Journal of cell science.

[30] Bracken AP, Dietrich N, Pasini D, Hansen KH, Helin K (2006). Genome-wide mapping of Polycomb target genes unravels their roles in cell fate transitions. Genes & development.

[31] Kwok HF, Zhang SD, McCrudden CM, Yuen HF, Ting KP, Wen Q, Khoo US, Chan KY (2015). Prognostic significance of minichromosome maintenance proteins in breast cancer. American journal of cancer research.

[32] Laskey R (2005). The Croonian Lecture 2001 hunting the antisocial cancer cell: MCM proteins and their exploitation. Philosophical transactions of the Royal Society of London Series B, Biological sciences.

[33] Ayaru L, Stoeber K, Webster GJ, Hatfield AR, Wollenschlaeger A, Okoturo O, Rashid M, Williams G, Pereira SP (2008). Diagnosis of pancreaticobiliary malignancy by detection of minichromosome maintenance protein 5 in bile aspirates. British journal of cancer.

[34] Liu DC, Yang ZL (2011). Clinicopathologic significance of minichromosome maintenance protein 2 and Tat-interacting protein 30 expression in benign and malignant lesions of the gallbladder. Human pathology.

[35] Srinivasan A, Gold B (2012). Small-molecule inhibitors of DNA damage-repair pathways: an approach to overcome tumor resistance to alkylating anticancer drugs. Future medicinal chemistry.

[36] Jalal S, Earley JN, Turchi JJ (2011). DNA repair: from genome maintenance to biomarker and therapeutic target. Clinical cancer research : an official journal of the American Association for Cancer Research.

[37] Kovalenko OV, Golub EI, Bray-Ward P, Ward DC, Radding CM (1997). A novel nucleic acid-binding protein that interacts with human rad51 recombinase. Nucleic acids research.

[38] Iwanaga R, Komori H, Ishida S, Okamura N, Nakayama K, Nakayama KI, Ohtani K (2006). Identification of novel E2F1 target genes regulated in cell cycle-dependent and independent manners. Oncogene.

[39] Henson SE, Tsai SC, Malone CS, Soghomonian SV, Ouyang Y, Wall R, Marahrens Y, Teitell MA (2006). Pir51, a Rad51-interacting protein with high expression in aggressive lymphoma, controls mitomycin C sensitivity and prevents chromosomal breaks. Mutation research.

[40] Obama K, Satoh S, Hamamoto R, Sakai Y, Nakamura Y, Furukawa Y (2008). Enhanced expression of RAD51 associating protein-1 is involved in the growth of intrahepatic cholangiocarcinoma cells. Clinical cancer research : an official journal of the American Association for Cancer Research.

[41] Wang M, Xiao J, Shen M, Yahong Y, Tian R, Zhu F, Jiang J, Du Z, Hu J, Liu W, Qin R (2011). Isolation and characterization of tumorigenic extrahepatic cholangiocarcinoma cells with stem cell-like properties. International journal of cancer Journal international du cancer.

[42] Keeratichamroen S, Leelawat K, Thongtawee T, Narong S, Aegem U, Tujinda S, Praditphol N, Tohtong R (2011). Expression of CD24 in cholangiocarcinoma cells is associated with disease progression and reduced patient survival. International journal of oncology.

[43] Iwahashi S, Utsunomiya T, Shimada M, Saito Y, Morine Y, Imura S, Ikemoto T, Mori H, Hanaoka J, Bando Y (2013). High expression of cancer stem cell markers in cholangiolocellular carcinoma. Surg Today.

[44] Leelawat K, Keeratichamroen S, Leelawat S, Tohtong R (2013). CD24 induces the invasion of cholangiocarcinoma cells by upregulating CXCR4 and increasing the phosphorylation of ERK1/2. Oncology letters.

[45] Shuang ZY, Wu WC, Xu J, Lin G, Liu YC, Lao XM, Zheng L, Li S (2014). Transforming growth factor-beta1-induced epithelial-mesenchymal transition generates ALDH-positive cells with stem cell properties in cholangiocarcinoma. Cancer letters.

[46] Douville J, Beaulieu R, Balicki D (2009). ALDH1 as a functional marker of cancer stem and progenitor cells. Stem cells and development.

[47] Wu A, Luo W, Zhang Q, Yang Z, Zhang G, Li S, Yao K (2013). Aldehyde dehydrogenase 1, a functional marker for identifying cancer stem cells in human nasopharyngeal carcinoma. Cancer letters.

[48] Charafe-Jauffret E, Ginestier C, Bertucci F, Cabaud O, Wicinski J, Finetti P, Josselin E, Adelaide J, Nguyen TT, Monville F, Jacquemier J, Thomassin-Piana J, Pinna G, Jalaguier A, Lambaudie E, Houvenaeghel G (2013). ALDH1-positive cancer stem cells predict engraftment of primary breast tumors and are governed by a common stem cell program. Cancer research.

[49] Ginestier C, Hur MH, Charafe-Jauffret E, Monville F, Dutcher J, Brown M, Jacquemier J, Viens P, Kleer CG, Liu S, Schott A, Hayes D, Birnbaum D, Wicha MS, Dontu G (2007). ALDH1 is a marker of normal and malignant human mammary stem cells and a predictor of poor clinical outcome. Cell stem cell.

[50] Charafe-Jauffret E, Ginestier C, Birnbaum D (2009). Breast cancer stem cells: tools and models to rely on. BMC Cancer.

[51] Mayr C, Wagner A, Stoecklinger A, Jakab M, Illig R, Berr F, Pichler M, P DIF, Ocker M, Neureiter D, Kiesslich T (2015). 3-Deazaneplanocin A May Directly Target Putative Cancer Stem Cells in Biliary Tract Cancer. Anticancer research.

[52] Oertel M, Schastak SI, Tannapfel A, Hermann R, Sack U, Mossner J, Berr F (2003). Novel bacteriochlorine for high tissue-penetration: photodynamic properties in human biliary tract cancer cells *in vitro* and in a mouse tumour model. Journal of photochemistry and photobiology B, Biology.

[53] Shimizu Y, Demetris AJ, Gollin SM, Storto PD, Bedford HM, Altarac S, Iwatsuki S, Herberman RB, Whiteside TL (1992). Two new human cholangiocarcinoma cell lines and their cytogenetics and responses to growth factors, hormones, cytokines or immunologic effector cells. International journal of cancer Journal international du cancer.

[54] Scherdin G, Garbrecht M, K M (1987). *In vitro* interaction of a-difluoromethylornithine (DFMO) and human recombinant interferon-a (rIFN-a) on human cancer cell lines. Immunobiology.

[55] Knuth A, Gabbert H, Dippold W, Klein O, Sachsse W, Bitter-Suermann D, Prellwitz W, Meyer zum Buschenfelde KH (1985). Biliary adenocarcinoma. Characterisation of three new human tumor cell lines. Journal of hepatology.

[56] Saijyo S, Kudo T, Suzuki M, Katayose Y, Shinoda M, Muto T, Fukuhara K, Suzuki T, Matsuno S (1995). Establishment of a new extrahepatic bile duct carcinoma cell line, TFK-1. The Tohoku journal of experimental medicine.

[57] Purdum PP, Ulissi A, Hylemon PB, Shiffman ML, Moore EW (1993). Cultured human gallbladder epithelia. Methods and partial characterization of a carcinoma-derived model. Laboratory investigation; a journal of technical methods and pathology.

[58] de Groen PC, Gores GJ, LaRusso NF, Gunderson LL, Nagorney DM (1999). Biliary tract cancers. N Engl J Med.

[59] Wachter J, Neureiter D, Alinger B, Pichler M, Fuereder J, Oberdanner C, Di Fazio P, Ocker M, Berr F, Kiesslich T (2012). Influence of five potential anticancer drugs on wnt pathway and cell survival in human biliary tract cancer cells. International journal of biological sciences.

[60] Kiesslich T, Alinger B, Wolkersdorfer GW, Ocker M, Neureiter D, Berr F (2010). Active Wnt signalling is associated with low differentiation and high proliferation in human biliary tract cancer *in vitro* and *in vivo* and is sensitive to pharmacological inhibition. International journal of oncology.

[61] Kiesslich T, Neureiter D, Alinger B, Jansky GL, Berlanda J, Mkrtchyan V, Ocker M, Plaetzer K, Berr F (2010). Uptake and phototoxicity of meso-tetrahydroxyphenyl chlorine are highly variable in human biliary tract cancer cell lines and correlate with markers of differentiation and proliferation. Photochemical & photobiological sciences : Official journal of the European Photochemistry Association and the European Society for Photobiology.

[62] Mayr C, Wagner A, Neureiter D, Pichler M, Jakab M, Illig R, Berr F, Kiesslich T (2015). The green tea catechin epigallocatechin gallate induces cell cycle arrest and shows potential synergism with cisplatin in biliary tract cancer cells. BMC complementary and alternative medicine.

[63] Livak KJ, Schmittgen TD (2001). Analysis of relative gene expression data using real-time quantitative PCR and the 2(−Delta Delta C(T)) Method. Methods.

[64] Stadnisky MD, Quinn J (2012). Flow Cytometry Analysis Automation and Cloud Integration with FlowJo Version 10. Genetic Engineering News.

