# Supplementary material for: The BMI1 inhibitor PTC-209 is a potential compound to halt cellular growth in biliary tract cancer cells
Source: Oncotarget. 2015 Nov 24;7(1):745–58. doi: 10.18632/oncotarget.6378 (PMC4808030; doi:10.18632/oncotarget.6378)
Supplement: Supplementary file 1 [file oncotarget-07-0745-s001.pdf]

The BMI1 inhibitor PTC-209 is a potential compound to halt cellular growth in biliary tract cancer cells

Supplementary Material

Cell line	PTC-209 (µM)										IC_{10} (µM)	IC_{50} (µM)
	20.0	10.00	5.00	2.50	1.25	0.63	0.31	0.16	0.08	0.04		
BDC		*	*	*	*						0.41	1.5
CCSW-1		*	*		*						1.04	n.q.
EGi-1											n.q.	n.q.
GBC	**	**	**	**	**	**	*	*			0.18	0.84
MzChA-1	*	**	**	**	**	**	*				0.17	0.51
MzChA-2		*	**	*	*	*					0.49	8.33
SkChA-1		*				*					0.25	1.44
TFK-1	**	**	**	**	**	**	*		*		0.24	n.q.

File name: Additional file 1

Title of data: Statistics for figure 2A

Description of data: Significant differences in cell viability between treated and untreated samples for the different cell lines and PTC-209 concentrations; 10% inhibitory concentration (IC10), 50% inhibitory concentration (IC50). Abbreviations: n.q.: not quantifiable

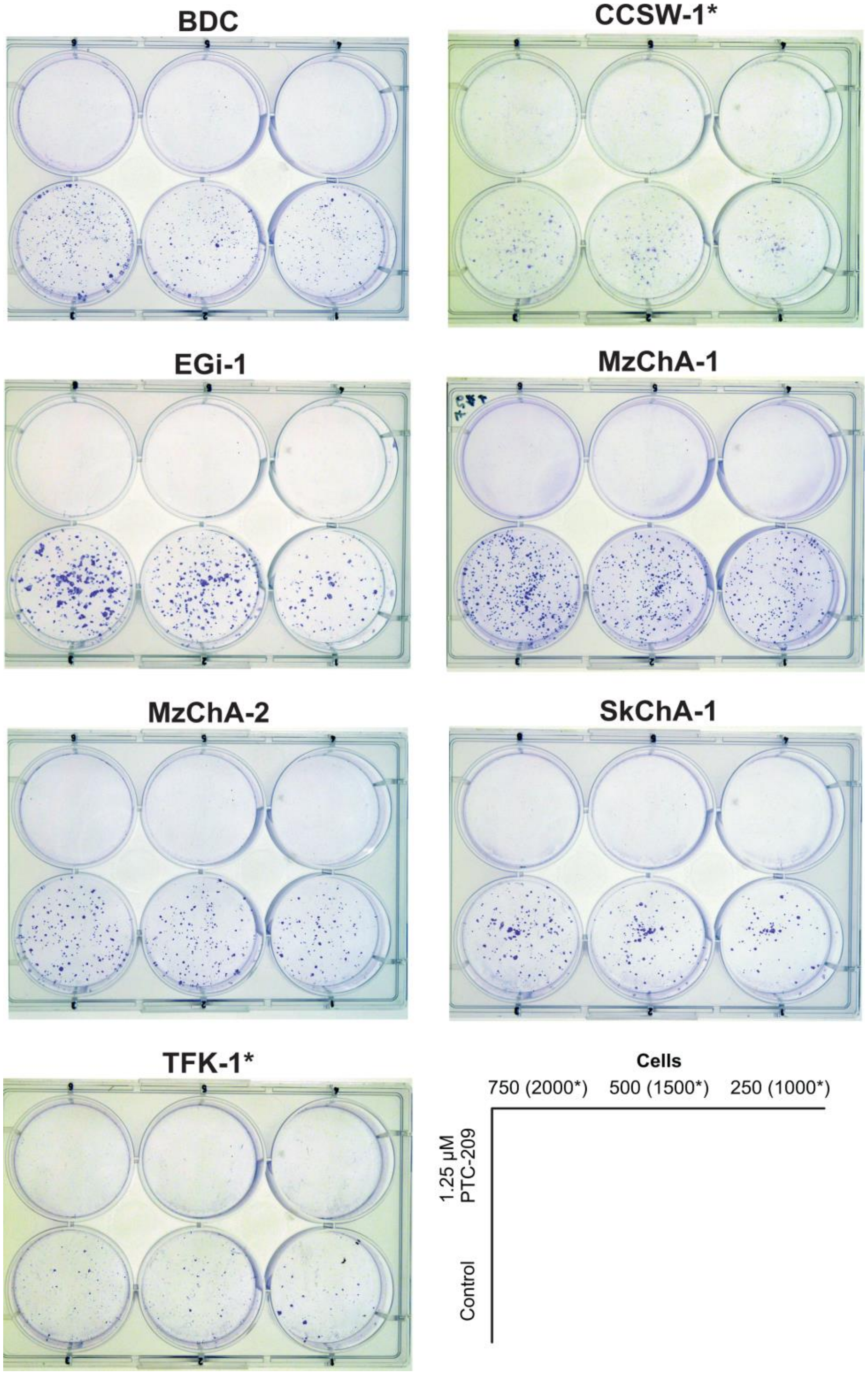

File name: Additional file 2

Title of data: Clonogenic assay for BTC cells

Description of data: BTC cell lines were treated with 1.25 µM PTC-209 or left untreated for 7 to 14 days, dependent on the cell line. Images of representative assays.

File name: Additional file 3
Title of data: PTC-209 halts cellular growth of biliary tract cancer cells
Description of data: Time course experiments were carried out on the JuLi BR live cell movie analyzer (NanoEnTek, Seoul, Korea). GBC cells were either treated with 1.25 µM PTC-209 in sfDMEM or left untreated (sfDMEM only) and cell growth was monitored in parallel on two microscopy units in a humidified cell culture incubator (37°C, 5% CO_2) by taking brigthfield microscopy images every five minutes for 72 h (865 images per sample).

Gene symbol	fold change	sem	Refseq	Description
ABL1	-1.39	0.191	NM_005157	C-abl oncogene 1, non-receptor tyrosine kinase
ANAPC2	-1.08	0.214	NM_013366	Anaphase promoting complex subunit 2
ATM	-1.33	0.277	NM_000051	Ataxia telangiectasia mutated
ATR	-1.35	0.176	NM_001184	Ataxia telangiectasia and Rad3 related
AURKA	1.94	0.079	NM_003600	Aurora kinase A
AURKB	-1.31	0.228	NM_004217	Aurora kinase B
BCCIP	-1.46	0.352	NM_016567	BRCA2 and CDKN1A interacting protein
BCL2	-3.39	0.582	NM_000633	B-cell CLL/lymphoma 2
BIRC5	1.97	0.066	NM_001168	Baculoviral IAP repeat containing 5
BRCA1	-3.76	0.551	NM_007294	Breast cancer 1, early onset
BRCA2	-1.98	0.333	NM_000059	Breast cancer 2, early onset
CASP3	-1.41	0.136	NM_004346	Caspase 3, apoptosis-related cysteine peptidase
CCNA2	1.82	0.188	NM_001237	Cyclin A2
CCNB1	2.90	0.769	NM_031966	Cyclin B1
CCNB2	2.39	0.424	NM_004701	Cyclin B2
CCNC	-1.40	0.107	NM_005190	Cyclin C
CCND1	-1.74	0.227	NM_053056	Cyclin D1
CCND2	1.50	0.242	NM_001759	Cyclin D2
CCND3	-2.32	0.048	NM_001760	Cyclin D3
CCNE1	-17.00	1.996	NM_001238	Cyclin E1
CCNF	1.23	0.115	NM_001761	Cyclin F
CCNG1	-2.86	0.563	NM_004060	Cyclin G1
CCNG2	1.80	0.423	NM_004354	Cyclin G2
CCNH	1.56	0.022	NM_001239	Cyclin H
CCNT1	1.71	0.184	NM_001240	Cyclin T1
CDC16	-1.42	0.181	NM_003903	Cell division cycle 16 homolog (S. cerevisiae)
CDC20	2.73	0.702	NM_001255	Cell division cycle 20 homolog (S. cerevisiae)
CDC25A	-5.72	1.369	NM_001789	Cell division cycle 25 homolog A (S. pombe)
CDC25C	1.88	0.154	NM_001790	Cell division cycle 25 homolog C (S. pombe)
CDC34	1.03	0.131	NM_004359	Cell division cycle 34 homolog (S. cerevisiae)
CDC6	-2.01	0.278	NM_001254	Cell division cycle 6 homolog (S. cerevisiae)
CDK1	1.14	0.087	NM_001786	Cyclin-dependent kinase 1
CDK2	-2.65	0.322	NM_001798	Cyclin-dependent kinase 2
CDK4	-2.50	0.214	NM_000075	Cyclin-dependent kinase 4
CDK5R1	-2.15	0.301	NM_003885	Cyclin-dependent kinase 5, regulatory subunit 1 (p35)
CDK5RAP1	1.35	0.145	NM_016408	CDK5 regulatory subunit associated protein 1
CDK6	1.20	0.184	NM_001259	Cyclin-dependent kinase 6
CDK7	1.22	0.172	NM_001799	Cyclin-dependent kinase 7
CDK8	-1.97	0.190	NM_001260	Cyclin-dependent kinase 8
CDKN1A	2.02	0.032	NM_000389	Cyclin-dependent kinase inhibitor 1A (p21, Cip1)
CDKN1B	1.71	0.338	NM_004064	Cyclin-dependent kinase inhibitor 1B (p27, Kip1)
CDKN2A	n.q.	n.q.	NM_000077	Cyclin-dependent kinase inhibitor 2A (melanoma, p16, inhibits CDK4)
CDKN2B	2.05	0.298	NM_004936	Cyclin-dependent kinase inhibitor 2B (p15, inhibits CDK4)
CDKN3	1.60	0.189	NM_005192	Cyclin-dependent kinase inhibitor 3
CHEK1	-2.08	0.278	NM_001274	CHK1 checkpoint homolog (S. pombe)
CHEK2	-1.23	0.156	NM_007194	CHK2 checkpoint homolog (S. pombe)
CKS1B	1.11	0.169	NM_001826	CDC28 protein kinase regulatory subunit 1B
CKS2	1.36	0.101	NM_001827	CDC28 protein kinase regulatory subunit 2
CUL1	-1.42	0.280	NM_003592	Cullin 1
CUL2	-1.57	0.219	NM_003591	Cullin 2
CUL3	1.12	0.132	NM_003590	Cullin 3
E2F1	-5.16	0.700	NM_005225	E2F transcription factor 1
E2F4	-1.15	0.053	NM_001950	E2F transcription factor 4, p107/p130-binding
GADD45A	2.02	0.219	NM_001924	Growth arrest and DNA-damage-inducible, alpha
GTSE1	2.96	0.232	NM_016426	G-2 and S-phase expressed 1
HUS1	-1.21	0.098	NM_004507	HUS1 checkpoint homolog (S. pombe)
KNTC1	-2.37	0.040	NM_014708	Kinetochore associated 1
KPNA2	1.13	0.069	NM_002266	Karyopherin alpha 2 (RAG cohort 1, importin alpha 1)
MAD2L1	1.17	0.097	NM_002358	MAD2 mitotic arrest deficient-like 1 (yeast)
MAD2L2	-1.50	0.213	NM_006341	MAD2 mitotic arrest deficient-like 2 (yeast)
MCM2	-8.55	0.633	NM_004526	Minichromosome maintenance complex component 2
MCM3	-7.23	0.594	NM_002388	Minichromosome maintenance complex component 3
MCM4	-2.95	0.459	NM_005914	Minichromosome maintenance complex component 4
MCM5	-7.19	1.223	NM_006739	Minichromosome maintenance complex component 5
MDM2	1.19	0.132	NM_002392	Mdm2 p53 binding protein homolog (mouse)
MKI67	1.21	0.030	NM_002417	Antigen identified by monoclonal antibody Ki-67
MNAT1	1.17	0.099	NM_002431	Menage a trois homolog 1, cyclin H assembly factor (Xenopus laevis)
MRE11A	-2.40	0.329	NM_005590	MRE11 meiotic recombination 11 homolog A (S. cerevisiae)
NBN	1.14	0.126	NM_002485	Nibrin
RAD1	-1.07	0.092	NM_002853	RAD1 homolog (S. pombe)
RAD17	1.25	0.143	NM_002873	RAD17 homolog (S. pombe)
RAD51	-2.56	0.384	NM_002875	RAD51 homolog (S. cerevisiae)
RAD9A	-1.91	0.327	NM_004584	RAD9 homolog A (S. pombe)
RB1	1.00	0.132	NM_000321	Retinoblastoma 1
RBBP8	-1.04	0.027	NM_002894	Retinoblastoma binding protein 8
RBL1	-2.14	0.423	NM_002895	Retinoblastoma-like 1 (p107)
RBL2	-1.45	0.131	NM_005611	Retinoblastoma-like 2 (p130)
SERTAD1	1.23	0.206	NM_013376	SERTA domain containing 1
SKP2	-3.70	0.446	NM_005983	S-phase kinase-associated protein 2 (p45)
STMN1	1.51	0.210	NM_005563	Stathmin 1
TFDP1	-3.31	0.331	NM_007111	Transcription factor Dp-1
TFDP2	-4.28	0.612	NM_006286	Transcription factor Dp-2 (E2F dimerization partner 2)
TP53	1.25	0.215	NM_000546	Tumor protein p53
WEE1	1.28	0.239	NM_003390	WEE1 homolog (S. pombe)

File name: Additional file 4

File format: pdf

Title of data: Supplementary data for figure 3C

Description of data: Complete list of all 84 cell cycle related genes investigated for changes in expression following PTC-209 treatment.

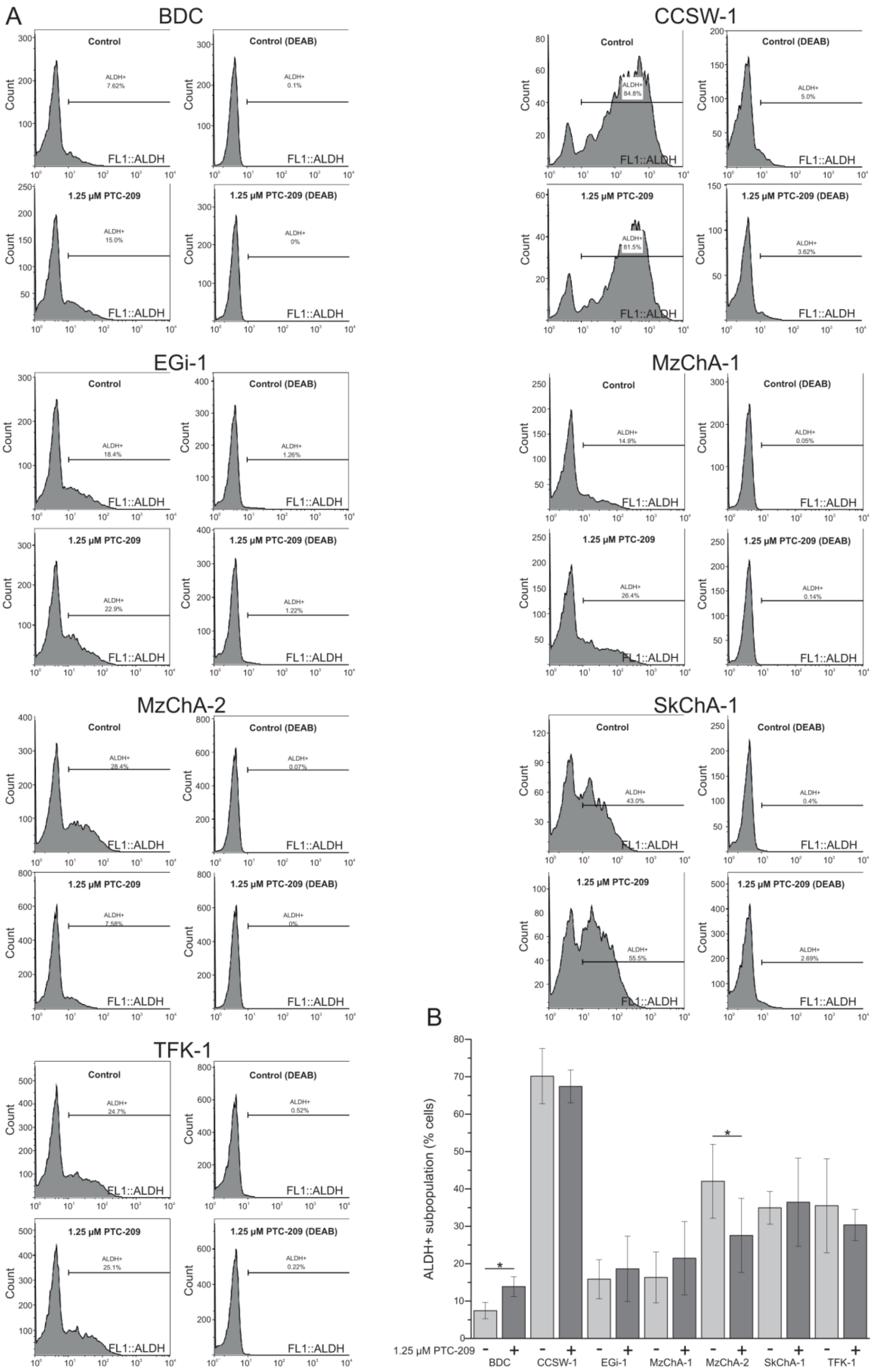

A
BDC
Control
ALDH+ 7.62%
Control (DEAB)
ALDH+ 0.1%
1.25 µM PTC-209
ALDH+ 15.0%
1.25 µM PTC-209 (DEAB)
ALDH+ 0%
CCSW-1
Control
ALDH+ 84.8%
Control (DEAB)
ALDH+ 5.0%
1.25 µM PTC-209
ALDH+ 81.5%
1.25 µM PTC-209 (DEAB)
ALDH+ 3.62%
EGi-1
Control
ALDH+ 18.4%
Control (DEAB)
ALDH+ 1.26%
1.25 µM PTC-209
ALDH+ 22.9%
1.25 µM PTC-209 (DEAB)
ALDH+ 1.22%
MzChA-1
Control
ALDH+ 14.9%
Control (DEAB)
ALDH+ 0.05%
1.25 µM PTC-209
ALDH+ 26.4%
1.25 µM PTC-209 (DEAB)
ALDH+ 0.14%
MzChA-2
Control
ALDH+ 28.4%
Control (DEAB)
ALDH+ 0.07%
1.25 µM PTC-209
ALDH+ 7.58%
1.25 µM PTC-209 (DEAB)
ALDH+ 0%
SkChA-1
Control
ALDH+ 43.0%
Control (DEAB)
ALDH+ 0.4%
1.25 µM PTC-209
ALDH+ 55.5%
1.25 µM PTC-209 (DEAB)
ALDH+ 2.69%
TFK-1
Control
ALDH+ 24.7%
Control (DEAB)
ALDH+ 0.52%
1.25 µM PTC-209
ALDH+ 25.1%
1.25 µM PTC-209 (DEAB)
ALDH+ 0.22%
Count
FL1::ALDH
B
ALDH+ subpopulation (% cells)
1.25 µM PTC-209
BDC
CCSW-1
EGi-1
MzChA-1
MzChA-2
SkChA-1
TFK-1

File name: Additional file 5

Title of data: Effect of PTC-209 on ALDH+ subpopulation in BTC cell lines

Description of data: Cells were treated with 1.25 µM PTC-209 or left untreated (sfDMEM only) for 72 h. (A) Representative histograms for each cell line including negative DEAB control. (B) Quantification of effect of PTC-209 treatment on ALDH+ subpopulation in BTC cells. Abbreviations: ALDH+: aldehyde dehydrogenase-1 positive cells; BTC: biliary tract cancer; DEAB: N,N-diethylaminobenzaldehyde; h: hours
